# Role of plasma homocysteine levels and other associated factors with coronary artery disease among Palestinian patients in North Palestine: a case control study

**DOI:** 10.11604/pamj.2022.42.180.34264

**Published:** 2022-07-06

**Authors:** Issa Alawneh, Abulkareem Saymeh, Muath Daraghmeh, Dima Jabri, Liana Yaseen

**Affiliations:** 1 Department of Pediatrics, Najah University Hospital, Nablus, Palestine,; 2 Department of Radiology, Rafedia Hospital, Nablus, Palestine,; 3 Faculty of Medicine and Health Sciences, Najah National University, Nablus, Palestine

**Keywords:** Coronary artery disease, homocysteine, smoking

## Abstract

**Introduction:**

coronary artery disease (CAD) is one of the leading causes of death worldwide, only two thirds of cases can be explained by CAD´s classical risk factors. There is an increase in attention to homocysteine as a causal of CAD. In Palestine, CAD is more common than that in regional surrounding areas. Coronary artery disease is considered the leading cause of death in the West Bank, according to Palestinian Ministry of health annual reports. The study was conducted to determine the level of homocysteine in individuals with no history of CAD and to determine the relationship between CAD and total homocysteine levels and classical risk factors of CAD.

**Methods:**

our study is a hospital-based case-control study. A sample size of 84 cases and 81 controls were included in the study.

**Results:**

there is a statistically significant increase in plasma total homocysteine level in cases compared to controls (P=0.04) with Odds ratio= 2. There is also a statistically significant association between plasma total homocysteine levels and age of 50 years and above and male gender among both study groups (P= 0.002 and 0.007, respectively). The study showed no significant association between plasma total homocysteine level and the CAD´s classical risk factors among the case group measured in the study, which are diabetes mellitus, hypertension, and smoking (P=0.5, 0.1, and 0.5, respectively).

**Conclusion:**

there is a significant difference in homocysteine levels between case and control groups. Coronary artery disease patients have double homocysteine levels compared to those healthy individuals with OR= 2. Healthy Palestinian individuals have a homocysteine level that is lower than that of regional communities. Homocysteine levels increase with advanced age and male gender. There is no significant relation between hyperhomocysteinemia and diabetes mellitus, hypertension, and smoking.

## Introduction

Coronary artery disease (CAD) is one of the main health problems concerning health care services worldwide. In recent years, attempts to combat this disease have extended beyond treatment and have centered mainly on prevention [1]. Despite steady progress in the treatment of cardiovascular diseases, people are still dying of these diseases, mainly at later ages [2]. In recent years, it has been suggested that only one-half to two-thirds of risks for atherosclerotic vascular disease can be explained by classic risk factors, like smoking, obesity, hypercholesterolemia, family history, physical inactivity, and diabetes mellitus, hypertension, and other co-morbidities [3]. Other risk factors that have come under scrutiny for their potential contribution include estrogen deficiency, lipoprotein (a), plasma fibrinogen, plasminogen activator inhibitor type I, endogenous tissue plasminogen activator (tPA), C-reactive protein and homocysteine [4]. There is a growing recognition that a high level of homocysteine is associated with heart disease [5]. According to a 1999 science advisory from american heart association (AHA) nutrition committee, plasma concentrations of fasting homocysteine between 5 and 15µmol/L are considered normal [6]. Elevated homocysteine levels are referred to as hyperhomocysteinemia, moderate: between 16 and 30 µmol/L; intermediate: between 31 and 100 µmol/L and severe: higher than 100 µmol/l [6]. Plasma homocysteine is increasingly recognized as an independent risk factor for vascular disease including coronary artery disease, cerebrovascular disease, and peripheral vascular diseases as well as deep venous thrombosis [7].

The first useful meta-analysis, by Boushey *et al*., showed that an increase in homocysteine levels of 5 µmol/L raises the relative risk for CAD by the same amount as does an increase in total cholesterol levels of 20mg/Dl Boushey *et al*. [8]. A positive relationship between homocysteine and the risk of myocardial infarction was seen in a large prospective community study from Norway [9]. Schnyder *et al*. [10], showed that total homocysteine together with age and gender is a strong predictor of the severity of coronary artery disease. The mortality rate has been reported to be significantly increased among patients of CAD who had high total homocysteine levels; for example, after 4 years of follow-up, the Kaplan-Meier estimates of mortality were 3.8% for patients with total homocysteine levels <9 µmol/L, 8.6% for those with levels of 9-14.9 µmol/L, and 24.7% for those with levels of >15 µmol/L [11]. In large European concerted action project which involved 750 patients with arterial vascular disease and 800 controls, confirmed that an elevated plasma homocysteine level was an independent risk factor for cardiovascular disease and calculated that an increase of 5 µmol/L in fasting basal homocysteine level was associated with a relative risk for cardiovascular disease of 1.35 in men and 1.42 in women [12]. In Palestine, CAD is the leading cause of death according to the ministry of health (MOH) annual report in 2009 [13]. Regarding MOH annual report in 2005, the mortality rate due to heart diseases was higher in West Bank compared to that of Gaza Strip [14]. There was a study done by Ramy Abu Sedo in 2012 in Gaza Strip showed that the mean levels of homocysteine were significantly higher in CAD patients compared to healthy individuals [15]. The current study is the first to investigate this issue in West Bank. The objectives of this study were to determine the relationship between CAD and total homocysteine level among patients in Nablus cardiac centers; (Arab specialized and Nablus specialized hospitals). In addition, to determine the level of homocysteine among individuals with no history of CAD in the above-mentioned centers. Moreover, to determine homocysteine levels among CAD patients in the above-mentioned centers. Indeed, to determine the relationship between levels of homocysteine and other CAD risk factors.

## Methods

**Study design:** this study is a hospital-based case-control study. Patients were recruited from the cardiology centers of Arab specialized Hospital and Nablus Specialized Hospital from the first of January 2013 to the 10^th^ of February 2013 and were assigned to the case group through the identification of cardiac catheterizations representative of at least 50% stenosis of at least one major coronary artery. A control was chosen from the same health centers with no history of CAD in the same time period.

**Study setting and population:** Arab specialized Hospital and Nablus Specialized Hospital, both are in Nablus, these two hospitals are considered as referral medical centers for most patients who will undergo coronary angiography in northern areas of the West Bank. Both cases and controls were recruited from these centers. The case group represented all patients admitted to the cardiology centers in the above-mentioned health centers and met the eligibility criteria. The control group represented patients admitted to the above-mentioned hospitals or reviewed for routine laboratory checkups with no history of coronary artery diseases. The inclusion criteria: For cases: patients of any age, having coronary artery disease (stenosis of at least 50 percent of the vessel diameter in at least one of the main coronary arteries) at the time of admission. Fasting for 4-6 hours. On the other hand, inclusion criteria for controls: any matching age groups referring to health centers. Fasting for 4-6 hours, no history of CAD. Cases or control groups who are eligible for participation in the study were excluded when they had one of the following: renal insufficiency (creatinine µ1.5 mg/DL). Liver failure [16]. Vitamin supplementation (Vit B12 and Vit B6, for more than five days a week in the previous three months) [17]. Fasting less than 4 hours.

### Data resource and measurement

**Data collection tool:** a special kit was used for measuring the plasma homocysteine level, which is a Homocysteine liquid-ultra vilet reagent kit, 40ml, enzymatic colorimetric test (334nm, 340nm, 365nm) its Reference No. is 11140 and produced by human diagnostics, Germany. A fully automated clinical chemistry analyzer was used for sample analysis (Miura one, Italy).

**Data collection:** an Arabic information sheet about the study was given to the study subjects, and then informed consent was taken from them. A meeting interview was used for filling especially designed data collection form for both case and control groups. All interviews were conducted face to face by the researchers themselves. During the survey, the interviewer explained any of the questions that were not clear. Most questions were yes/no questions, which offers a dichotomous choice. It provided information about the personal information (age, gender, and smoking), socioeconomic characteristics (family history of CAD), clinical data (previous myocardial infarction, hypertension, diabetes mellitus, renal impairment -dialysis, liver failure, vitamin supplementation; B12 and B6). A pilot study was done prior to the beginning of real data collection to know the length and clarity of the data collection form and to evaluate the outcome. Ten cases were interviewed. At the end of the pilot study, a comprehensive revision to the questionnaire was made and modified as necessary. The pilot subjects were not included in the study. A venous blood sample from both the case and control groups was collected by nurse staff in the above-mentioned centers after at least (4 to 6)-hours of fasting into tubes containing ethylenediamine tetraacetic acid (EDTA). Blood samples were immediately sent to laboratories in the same hospitals for plasma separation obtained by centrifugation at 3000rpm/10 minutes, within 30 minutes of blood collection, and kept frozen there at -21 Celsius [15] till the time of transfer. Plasma samples were delivered to the Genetic laboratory at An-Najah National University on dry ice for total homocysteine level measurements later. Samples were frozen at -21 Celsius [15]. The samples have been analyzed at An-Najah National Educational Hospital´s Laboratory.

**Sample size:** the total number of cases was calculated depending on the special equation for the case-control study sample size, as shown below [18]. The standard deviation of homocysteine level was set as 13.9 [19], and the least significant mean difference between cases and controls was set as 6 µmol/Las mentioned earlier in Boushey *et al*. meta-analysis [8]. This results in a sample size of 84 cases.


$$n=(\frac{r+1}{r})\frac{\sigma^{2}{\left(Z_{\beta }+Z_{\alpha /2}\right)}^{2}}{{\left(difference\right)}^{2}}$$

For a power of 80%, Z_β_is 0.84 and for a significance level α of 0.05, Z_α_is 1.96. In addition, r=1 which is the ratio of cases to controls; σ =13.9, and the difference (difference between the mean of homocysteine between case and control) =6. A similar number of controls were recruited (84) with a case-to-control ratio equal 1: 1. Although recruiting more controls may conclude better results, the constraints of cost and time make the ratio 1: 1 reasonable.

**Data analysis:** all statistical analysis was carried out using the Statistical Package for Social Sciences (SPSS V.17 SPSS Inc., Chicago, Illinois, USA). Descriptive statistics were done by summarizing continuous variables as mean and standard deviations, and categorical variables by frequencies and percentages. Chi-square test was used to compare categories of categorical variables between cases and controls; an independent samples t-test was used to compare the mean ages between cases and controls, Mann-Whitney test was used to compare medians of total homocysteine levels as they didn´t show a normal distribution when normality test was done. The level of significance α was set as 0.05 so p-value was considered significant when it is< 0.05.

**Ethical issues:** we have taken the permission to carry out this study from An-Najah National University Institutional Review Board (IRB), Ministry of Health, Arab Specialized Hospital, Nablus specialized Hospital, and An-Najah National Educational Hospital´s Laboratories. Informed written consent had been taken from the participants. Participants had been assured that all data collected will be confidential. The data collected are used for research purposes only.

**Data availability:** the datasets generated and analyzed during the current study are not publicly available due to participant private policies and research regulation agreements related to Najah National University, but are available from the corresponding author on reasonable request.

## Results

**Characteristics of the sample:** a number of 95 cases and 90 control subjects were interviewed, and their agreements were taken to participate in the study. Eleven cases and nine controls were excluded from the study due to various reasons such as the presence of missed data, blood sample volume was not enough or technical errors during centrifugation. A final total of 84 cases and 81 controls were included in the study. Table 1 summarizes the demographic and clinical characteristics of study participants. The mean age of cases was found to be 57.63±11.1 years (n=84). While the mean age of controls X was 46.23±14.65 years (n=84). Our data showed that cases had more males than controls (Table 1). Both cases and controls have the same percentages of smokers. Cases participants were shown to have diabetes, hypertension and a family history of MI compared to control (Table 1). However, statistical analysis showed no significant association between having CAD and either hypertension or a family history of MI.

**Table 1:** main demographic and clinical characteristics of study samples

Variable	Case group (n=84) N (%)	Control group (n=81) N (%)	P-Value
Age y mean ± SD a	57.63 ±11.1	46.23 ± 14.65	p <0.001*
Male b	65 (77.4%)	50 (61.7%)	P= 0.029*
Current smoker b	35 (42.2%)	34 (42%)	P = 0.98
Hypertension b	35 (41.7%)	25 (30.9%)	P = 0.15
Diabetes mellitus b	34 (41%)	23 (28.4%)	P = 0.09
Family history of MI b	27 (32.1%)	17 (21%)	P = 0.105

**Homocysteine level and coronary artery disease:** homocysteine levels among cases and controls have been determined (Table 2). The median total homocysteine level among the control group was 8.57µmol/L. The level however among the case group was 10.83 µmol/L. Of 84 cases 20 patients have abnormal total homocysteine level of 15 µmol/L or more (23.8%), while of 81 controls 11 only have a total homocysteine level of 15 µmol/L or more (13.6%). By using the Mann-Whitney test, there was a statistically significant difference between cases and controls with regard to homocysteine level (U=2949.5, P=0.04). The odds ratio was calculated and found to be 2 (OR=2).

**Table 2:** homocystein level among Palestinians suffering from coronary artery disease (cases) and healthy individuals with no history of CAD (controls)

	Case group	Control group	p-value
Mean homocysteine level	11.91±7.63	9.79±5.74	
Median homocysteine level	10.83	8.57	P = 0.04*

Mann-Whitney test was used; *Statistically significant ,α=0.05

**Homocysteine level and gender:** in both study groups, the median total homocysteine level among males (n=115) was 10.8 while in females (n=50) was 7.23 with a statistically significant increase of total homocysteine level in males compared to females in both study groups using the Mann-Whitney test (U=2205, P=0.007).

**Homocysteine level and age:** the median homocysteine level among people with age less than 50 years in both study groups was 8.5 while in people with age of 50 years and above was 10.8, by using the Mann-Whitney test, there was a statistically significant increase in homocysteine level in people with age 50 and above in both study groups compared to people with age less than 50 (U=2539, P=0.002).

**Homocysteine level and classical risk factors of CAD:** we classified participants (both cases and controls) according to the presence or absence of the classical CAD risk factors.

**Homocysteine level and diabetes mellitus:** the median total homocysteine level among diabetics in both study groups was 10.7 while in the non-diabetics was 9.5 with no significant statistical increase in total homocteine level in diabetics in both study groups (U=3082, P=0.5). In the case group, the median homocyateine level among diabetics was 10.8 while in non-diabetics was 11. By using the Mann-Whitney test, there was no statistically significant increase in total homocystein level among diabetics compared to non-diabetics in the case group (U=884, P=0.5).

**Homocysteine level and hypertension:** the median homocysteine level among hypertensives in both study groups was 10.8 while in non-hypertensives was 9.6 with statistically no significant increase in total homocysteine level among hypertensive sample in both study groups (U=2923, P=0.13). In the case of a group, the median homocyateine level among hypertensives was 11.6 while in non-hypertensives was 10.6. By using the Mann-Whitney test, there was no statistically significant increase in total homocystein level among hypertensives compared to non-hypertensives in the case group (U=790, P=0.1).

**Homocysteine level and smoking:** the median homocysteine level among smokers in both study groups was 10.9 while in non-smokers was 8.9 with statistically no significant increase in total homocteine level among smokers in both study groups (U=3181, P= 0.3). In the case of group, the median homocyateine level among smokers was 11.2 while in non-smokers was 10.2. By using the Mann-Whitney test, there was no statistically significant increase in total homocystein level among smokers compared to non-smokers in the case group (U=880, P=0.5) Table 3.

**Table 3:** total plasma homocystein level between coronary artery disease risk factors categories among case group

Diabetes mellitus	Yes	No	P value
mean Hcy level ± SD	11.3 ± 7.22	10 ± 6.4	
Median Hcy level	10.8	11	p =0.5
**Hypertension**	**Yes**	**No**	**P value**
Mean Hcy level± SD	12 ± 7.5	10.2 ± 6	
Median Hcy level	11.6	10.6	p =0.1
Smoking*	**Yes**	**No**	**P value**
mean Hcy level ±SD	11.2 ± 6.3	10.7 ± 7	
Median Hcy level	11.2	10.2	p =0.5

## Discussion

This study is the first of its type in North Palestine to the best of the author´s knowledge. It is very important to elucidate the association between homocysteine levels and the incidence of coronary artery disease (CAD) among Palestinians. It is evident from several observations the high incidence of myocardial infarction among young individuals. There are of course a number of risk factors, including both acquired and genetic conditions, associated with the development of CAD. Homocysteine level is associated with increased mortality among patients who have CAD. This study highlights the association between homocysteine levels and acute myocardial infarction. Case group has more males than the control, this is expected as the male gender is a risk factor for CAD, whereas female patients were less compliant during the study. This is reported in previous studies, which included more male patients than female patients. Herrmann *et al*. [7] showed a significantly high number of males in both cases and controls with p<0.001. Similarly, Lin *et al*. [20] reported that about 90% of the case group were males, compared to 55% of males in the control group. Age is found to have a significant association with the development of CAD among the study group. Our data showed that cases are older than controls (p<0.001) This is expected, as age is a well-known risk factor for developing atherosclerosis and CAD. Many studies reported differences in the mean age between case and control groups, such as; Eftychiou *et al*. [16] and Herrman *et al*. [7]. Our study showed that there was no significant difference between case and control groups regarding smoking status, hypertension, and diabetes mellitus. Many studies found significant differences regarding most of the classical risk factors, on the other hand, some studies reported significant differences only in one of them, Eftychiou *et al*. [16] reported a statically significant difference regarding hypertension but not to smoking or diabetes mellitus, this may be due to the fact that hypertension is more common in our community.

The median level of total homocysteine in non-CAD individuals in the North West Bank, which is 8.57 µmol/L, appears to be lower than that of regional areas, like in the Syrian population (11 µmol/L), and that of Europeans, Germany as an example (8.8 µmol/L) [7]. On the other hand, the mean level of Homocysteine levels in CAD patients, which is 11.9µmol/L, is somewhat higher than that of Canadians (11.7µmol/L) [21] and Norway patients (11.4µmol/L) [11]. As mentioned in this study, there was a significant elevation in the level of homocysteine in cases compared to controls. This may conclude that a high homocysteine level is associated with CAD. This finding is in agreement with other reports elsewhere by Boushey *et al*. [8] and Arnesen *et al*. [9]. Several possible mechanisms for the association between homocysteine and atherosclerosis have been demonstrated in experimental models. These include stimulation of smooth muscle growth, reduction in endothelial cell growth, and impaired endothelial cell relaxation [15]. Women have lower total homocysteine than men. Additionally, an increase in total homocysteine has been noted in older people. hese observations could be explained by the effect of sex hormones [1]. In our study population, there were statically significant increments of total Homocysteine level in men compared to women. Our study data showed that there is no significant relationship between hyper-homocysteinemia and classical risk factors for CAD (Hypertension, DM, Smoking). This is in agreement with reports by Sundström *et al*. [22] and Guldener *et al*. [21] showed no relationship between the level of homocysteine, hypertension, and diabetes mellitus type 2, respectively. However, there are other studies that indeed found an association between homocysteine level and the above-mentioned risk factors [23,24].

**Limitations of the study:** i) delay in the provision of kits used for homocystein analysis; ii) most patients of CAD are males, and low compliance of female with the study; iii) cases are older than control groups.

## Conclusion

Level of total plasma homocysteine was significantly higher in cases compared to controls. In addition, homocysteine level was higher in individuals who are older than 50 years compared to those with younger ages. It was higher in male gender comparing to female. The level is not shown to be associated with other classical risk factors (Diabetes Mellitus, hypertension and smoking). Based on the results, it is recommended to follow up CAD patients with high homocysteine levels closely as this may decrease mortality.

### What is known about this topic

- CAD is one of the main health problems concerning health care services worldwide;
- In Palestine, CAD is the leading cause of death according to ministry of health annual report;
- Elevated level of homocysteine can be associated with CAD but a case control study was needed to prove the association.

### What this study adds

- Coronary artery disease patients have double homocysteine level compare to that of healthy individuals;
- Homocysteine levels increase with advanced age and male gender;
- There is no significant relation between hyperhomocysteinemia and diabetes mellitus, hypertension and smoking.
